# (*E*)-Ethyl 2-cyano-3-[5-nitro-2-(pyrrolidin-1-yl)phen­yl]acrylate

**DOI:** 10.1107/S1600536810033374

**Published:** 2010-09-04

**Authors:** Yapi Marcellin Yapo, Bakary Coulibaly Abou, Ané Adjou, Rita Kakou-Yao, Jules A. Tenon

**Affiliations:** a Laboratoire de Cristallographie et Physique Moléculaire, UFR–SSMT, Université de Cocody, 22 BP 582 Abidjan 22, Côte d’Ivoire; b Laboratoire de Chimie Organique Structurale, UFR–SSMT, Université de Cocody, 22 BP 582 Abidjan 22, Côte d’Ivoire

## Abstract

The title compound, C_16_H_17_N_3_O_4_, was prepared by the reaction of 5-nitro-2-(pyrrolidin-1-yl)benzaldehyde and ethyl cyano­acetate. The mol­ecular structure adopts an *E* conformation with respect to the C=C double bond. The five-membered ring has a half-chair conformation, with puckering parameters *Q*(2)= 0.399 (2) Å and ϕ = 93.1 (3)°. In the crystal, inversion dimers , linked by pairs of C—H⋯O inter­actions, are further connected through C—H⋯N hydrogen bonds. Weak slipped π-π inter­actions occur between symmetry-related benzene rings [centroid–centroid distance = 3.785 (1)Å].

## Related literature

For related structures, see: Yapo *et al.* (2010 ▶); Zhang *et al.* (2009*a* ▶,*b* ▶). For reference bond lengths, see: Allen (2002 ▶). For ring conformation analysis, see: Cremer & Pople (1975 ▶).
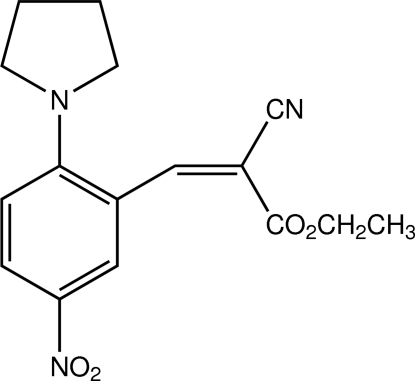


## Experimental

### 

#### Crystal data

C_16_H_17_N_3_O_4_
*M* *_r_* = 315.33
Triclinic, 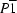

*a* = 8.4137 (3) Å
*b* = 9.9517 (4) Å
*c* = 10.3731 (5) Å
α = 73.065 (1)°
β = 71.388 (2)°
γ = 72.523 (4)°
*V* = 766.56 (6) Å^3^
*Z* = 2
Mo *K*α radiation
μ = 0.10 mm^−1^
*T* = 223 K
0.15 × 0.05 × 0.05 mm

#### Data collection

Nonius KappaCCD diffractometer
12261 measured reflections
3925 independent reflections
2436 reflections with *I* > 3σ(*I*)
*R*_int_ = 0.04

#### Refinement

*R*[*F* ^2^ > 2σ(*F* ^2^)] = 0.045
*wR*(*F* ^2^) = 0.098
*S* = 1.01
2436 reflections
208 parameters
H-atom parameters constrained
Δρ_max_ = 0.29 e Å^−3^
Δρ_min_ = −0.19 e Å^−3^

### 

Data collection: *COLLECT* (Nonius, 2001 ▶); cell refinement: *DENZO*/*SCALEPACK* (Otwinowski & Minor, 1997 ▶); data reduction: *DENZO*/*SCALEPACK*; program(s) used to solve structure: *SIR2004* (Burla *et al.*, 2005 ▶); program(s) used to refine structure: *CRYSTALS* (Betteridge *et al.*, 2003 ▶); molecular graphics: *ORTEPIII* (Burnett & Johnson, 1996 ▶), *ORTEP-3 for Windows* (Farrugia, 1997 ▶) and *PLATON* (Spek, 2009 ▶); software used to prepare material for publication: *CRYSTALS*.

## Supplementary Material

Crystal structure: contains datablocks global, I. DOI: 10.1107/S1600536810033374/dn2594sup1.cif

Structure factors: contains datablocks I. DOI: 10.1107/S1600536810033374/dn2594Isup2.hkl

Additional supplementary materials:  crystallographic information; 3D view; checkCIF report

## Figures and Tables

**Table 1:** Hydrogen-bond geometry (Å, °)

*D*—H⋯*A*	*D*—H	H⋯*A*	*D*⋯*A*	*D*—H⋯*A*
C15—H152⋯O2^i^	0.98	2.50	3.356 (2)	145
C16—H163⋯N3^ii^	0.98	2.60	3.574 (3)	172

Symmetry codes: (i) 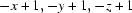; (ii) 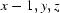.

**Fig. 1.**
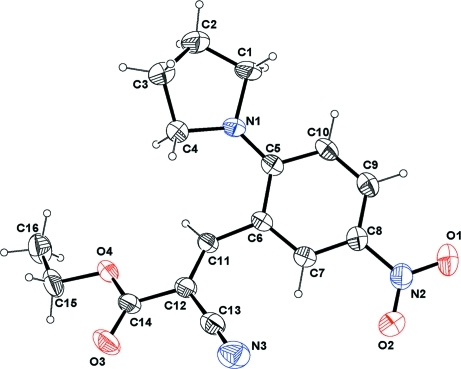
Molecular view of the title complex with the atom labeling scheme. Ellipsoids are drawn at the 50% probability level. H atoms are shown as small spheres of arbitary radii.

**Fig. 2.**
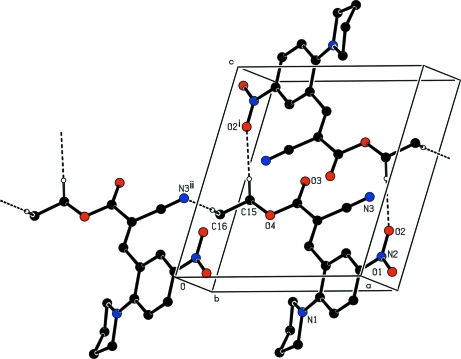
Partial packing view showing the hydrogen bond pattern. Hydrogen bonds are shown as dashed lines. H atoms not involved in hydrogen bondings have been omitted for clarity. [Symmetry codes: (i) -x+1, -y+1, -z+1; (ii) x-1, y, z ]

## supplementary crystallographic information

### Comment

Recently, the synthesis and structure of tricyclic quinoline derivative have been widely investigated (Yapo *et al.*, 2010). We report herein the crystal structure of the title compound C_16_H_17_N_3_O_4 _(I). In fact, it is an intermediate compound on which chemical reactions will be made to obtain tricyclic quinoline derivative containing in its molecular structure two coupled rings: quinoline ring and pyrrolidine ring.

The molecular structure of the title complex displays a E conformation with respect to the C11═C12 double bond. The bond distance C12— C13=1.436 (2)Å agrees with recently reported structures (Zhang *et al.*, 2009*a*; Zhang *et al.*, 2009*b*) and is characteristic of single bond occuring between carbone *sp*^1^ and carbone *sp*^2^ [C(*sp*^1^)—C(*sp*^2^)]. All other bond lengths and angles are not unusual (Allen, 2002).

The pyrrolidine ring has half-chair conformation with puckering parameters Q(2)= 0.399 (2)Å and φ= 93.1 (3)° (Cremer & Pople, 1975)

In the crystal packing, centrosymmetrically related molecules are linked by intermolecular C—H···O hydrogen bonding interactions building pseudo dimers which are further connected through C-H···N hydrogen bonds (Table 1 and Figure 2). Weak π-π interactions occur between symmetry related phenyl rings (Centroid-to-centroid = 3.785 (1)Å, interplanar distance= 3.509Å and a slippage of 1.417Å with symmetry code: (i) 2-x,1-y,-z).

### Experimental

To a solution of 5-nitro-2-(pyrrolidin-1-yl)benzaldehyde (2, 9.03 mmol) in anhydrous ethanol (25 ml), ethyl cyanoacetate (2.1 ml, 10.1 mmol) was added. Maintained at room temperature and under magnetic agitation, triethylamine (3 ml) was dropped into the solution. The reaction mixture was maintained at room temperature for 30 min then heated to ethanol reflux during 2 h. After cooling, the precipitate was filtred and then washed with ethanol to obtain yellow crystals in 77% yield. The melting point is 457–458 K.

### Refinement

The H atoms were geometrically positioned and treated as riding with C—H in the range 0.93–0.98Å and *U*_iso_(H) in the range 1.2–1.5 times *U*_eq_ of the parent atom.

### Crystal data

**Table:**

C_16_H_17_N_3_O_4_	*Z* = 2
*M**_r_* = 315.33	*F*(000) = 332
Triclinic, *P*1	*D*_x_ = 1.366 Mg m^−^^3^
Hall symbol: -P 1	Mo *K*α radiation, λ = 0.71073 Å
*a* = 8.4137 (3) Å	Cell parameters from 12261 reflections
*b* = 9.9517 (4) Å	θ = 4–29°
*c* = 10.3731 (5) Å	µ = 0.10 mm^−^^1^
α = 73.065 (1)°	*T* = 223 K
β = 71.388 (2)°	Prism, yellow
γ = 72.523 (4)°	0.15 × 0.05 × 0.05 mm
*V* = 766.56 (6) Å^3^	

### Data collection

**Table:**

Nonius KappaCCD diffractometer	*R*_int_ = 0.04
graphite	θ_max_ = 29.1°, θ_min_ = 2.1°
φ & ω scans	*h* = 0→11
12261 measured reflections	*k* = −12→13
3925 independent reflections	*l* = −12→14
2436 reflections with *I* > 3σ(*I*)	

### Refinement

**Table:**

Refinement on *F*^2^	Primary atom site location: structure-invariant direct methods
Least-squares matrix: full	Hydrogen site location: inferred from neighbouring sites
*R*[*F*^2^ > 2σ(*F*^2^)] = 0.045	H-atom parameters constrained
*wR*(*F*^2^) = 0.098	*w* = 1/[σ^2^(*F*^2^) + (0.04*P*)^2^ + 0.41*P*] where *P* = [max(*F*_o_^2^,0) + 2*F*_c_^2^]/3
*S* = 1.00	(Δ/σ)_max_ = 0.0002
2436 reflections	Δρ_max_ = 0.29 e Å^−^^3^
208 parameters	Δρ_min_ = −0.19 e Å^−^^3^
0 restraints	

### Fractional atomic coordinates and isotropic or equivalent isotropic displacement parameters (Å^2^)

**Table:**

	*x*	*y*	*z*	*U*_iso_*/*U*_eq_	
O4	0.24506 (16)	0.75552 (14)	0.34637 (12)	0.0392	
C6	0.6744 (2)	0.54044 (17)	0.08814 (16)	0.0272	
O3	0.38346 (18)	0.74040 (16)	0.50501 (13)	0.0501	
N1	0.58761 (18)	0.70320 (15)	−0.12042 (14)	0.0318	
C11	0.5357 (2)	0.61576 (17)	0.18686 (16)	0.0288	
C10	0.8072 (2)	0.48737 (19)	−0.14342 (18)	0.0349	
O2	1.03617 (19)	0.19215 (16)	0.22812 (15)	0.0536	
C14	0.3815 (2)	0.71447 (18)	0.39891 (17)	0.0335	
C5	0.6844 (2)	0.58161 (18)	−0.05898 (16)	0.0281	
C7	0.7910 (2)	0.41913 (18)	0.13881 (17)	0.0302	
C8	0.9117 (2)	0.33592 (18)	0.05070 (18)	0.0323	
N2	1.0334 (2)	0.21267 (17)	0.10625 (17)	0.0405	
C12	0.5355 (2)	0.63394 (17)	0.31088 (16)	0.0295	
O1	1.1330 (2)	0.13387 (17)	0.02781 (17)	0.0640	
C13	0.6834 (2)	0.5867 (2)	0.36710 (18)	0.0355	
N3	0.7983 (2)	0.5498 (2)	0.41549 (19)	0.0528	
C9	0.9177 (2)	0.3684 (2)	−0.09019 (18)	0.0361	
C1	0.6073 (3)	0.7384 (2)	−0.27297 (18)	0.0420	
C4	0.4900 (2)	0.83167 (19)	−0.06191 (18)	0.0374	
C16	−0.0507 (3)	0.8668 (2)	0.3594 (2)	0.0538	
C15	0.0917 (3)	0.8417 (3)	0.4249 (2)	0.0524	
C2	0.4880 (3)	0.8854 (2)	−0.2995 (2)	0.0520	
C3	0.4940 (3)	0.9553 (2)	−0.18931 (19)	0.0458	
H111	0.4299	0.6579	0.1601	0.0408*	
H101	0.8112	0.5097	−0.2402	0.0501*	
H71	0.7850	0.3922	0.2355	0.0416*	
H91	1.0005	0.3059	−0.1485	0.0499*	
H11	0.7291	0.7413	−0.3221	0.0611*	
H12	0.5771	0.6646	−0.3001	0.0612*	
H41	0.5454	0.8431	0.0025	0.0516*	
H42	0.3687	0.8243	−0.0126	0.0529*	
H161	−0.1516	0.9310	0.4046	0.0975*	
H162	−0.0169	0.9098	0.2594	0.0977*	
H163	−0.0794	0.7744	0.3742	0.0981*	
H151	0.1190	0.9366	0.4202	0.0740*	
H152	0.0663	0.7875	0.5221	0.0730*	
H21	0.5289	0.9403	−0.3927	0.0751*	
H22	0.3698	0.8790	−0.2855	0.0754*	
H31	0.6032	0.9898	−0.2180	0.0663*	
H32	0.3939	1.0346	−0.1743	0.0659*	

### Atomic displacement parameters (Å^2^)

**Table:**

	*U*^11^	*U*^22^	*U*^33^	*U*^12^	*U*^13^	*U*^23^
O4	0.0392 (7)	0.0457 (8)	0.0277 (6)	0.0026 (6)	−0.0057 (5)	−0.0162 (5)
C6	0.0280 (8)	0.0315 (9)	0.0230 (8)	−0.0089 (7)	−0.0040 (6)	−0.0078 (6)
O3	0.0563 (9)	0.0652 (10)	0.0332 (7)	−0.0074 (7)	−0.0099 (6)	−0.0257 (7)
N1	0.0394 (8)	0.0349 (8)	0.0223 (7)	−0.0071 (6)	−0.0101 (6)	−0.0070 (6)
C11	0.0311 (8)	0.0297 (9)	0.0234 (8)	−0.0071 (7)	−0.0044 (6)	−0.0054 (6)
C10	0.0393 (10)	0.0416 (10)	0.0250 (8)	−0.0098 (8)	−0.0041 (7)	−0.0132 (7)
O2	0.0579 (9)	0.0499 (9)	0.0435 (8)	0.0042 (7)	−0.0192 (7)	−0.0063 (6)
C14	0.0419 (10)	0.0331 (9)	0.0239 (8)	−0.0076 (7)	−0.0058 (7)	−0.0076 (7)
C5	0.0286 (8)	0.0332 (9)	0.0254 (8)	−0.0107 (7)	−0.0053 (6)	−0.0085 (7)
C7	0.0320 (9)	0.0330 (9)	0.0259 (8)	−0.0091 (7)	−0.0056 (7)	−0.0073 (7)
C8	0.0298 (9)	0.0312 (9)	0.0349 (9)	−0.0063 (7)	−0.0060 (7)	−0.0091 (7)
N2	0.0365 (9)	0.0372 (9)	0.0431 (9)	−0.0042 (7)	−0.0065 (7)	−0.0099 (7)
C12	0.0349 (9)	0.0290 (8)	0.0238 (8)	−0.0086 (7)	−0.0063 (7)	−0.0045 (6)
O1	0.0590 (9)	0.0562 (10)	0.0616 (10)	0.0174 (8)	−0.0105 (8)	−0.0274 (8)
C13	0.0427 (10)	0.0390 (10)	0.0266 (9)	−0.0133 (8)	−0.0060 (8)	−0.0086 (7)
N3	0.0521 (11)	0.0654 (12)	0.0484 (10)	−0.0155 (9)	−0.0213 (9)	−0.0120 (9)
C9	0.0359 (10)	0.0375 (10)	0.0345 (9)	−0.0089 (8)	−0.0015 (7)	−0.0149 (8)
C1	0.0566 (12)	0.0467 (11)	0.0233 (9)	−0.0082 (9)	−0.0149 (8)	−0.0069 (8)
C4	0.0435 (10)	0.0360 (10)	0.0300 (9)	−0.0026 (8)	−0.0103 (8)	−0.0090 (7)
C16	0.0431 (11)	0.0587 (14)	0.0599 (14)	−0.0028 (10)	−0.0097 (10)	−0.0260 (11)
C15	0.0436 (12)	0.0621 (14)	0.0447 (12)	0.0086 (10)	−0.0054 (9)	−0.0295 (10)
C2	0.0677 (14)	0.0514 (12)	0.0346 (10)	−0.0045 (10)	−0.0237 (10)	−0.0036 (9)
C3	0.0582 (13)	0.0381 (11)	0.0362 (10)	−0.0049 (9)	−0.0159 (9)	−0.0027 (8)

### Geometric parameters (Å, °)

**Table:**

O4—C14	1.330 (2)	C12—C13	1.436 (2)
O4—C15	1.462 (2)	C13—N3	1.145 (2)
C6—C11	1.460 (2)	C9—H91	0.963
C6—C5	1.442 (2)	C1—C2	1.511 (3)
C6—C7	1.394 (2)	C1—H11	0.993
O3—C14	1.207 (2)	C1—H12	0.982
N1—C5	1.350 (2)	C4—C3	1.520 (3)
N1—C1	1.483 (2)	C4—H41	0.976
N1—C4	1.477 (2)	C4—H42	0.999
C11—C12	1.350 (2)	C16—C15	1.483 (3)
C11—H111	0.955	C16—H161	0.971
C10—C5	1.426 (2)	C16—H162	0.983
C10—C9	1.363 (2)	C16—H163	0.977
C10—H101	0.955	C15—H151	1.021
O2—N2	1.227 (2)	C15—H152	0.984
C14—C12	1.484 (2)	C2—C3	1.521 (3)
C7—C8	1.377 (2)	C2—H21	0.972
C7—H71	0.948	C2—H22	0.977
C8—N2	1.448 (2)	C3—H31	1.003
C8—C9	1.389 (2)	C3—H32	0.972
N2—O1	1.233 (2)		
			
C14—O4—C15	114.97 (14)	N1—C1—H11	109.3
C11—C6—C5	121.29 (14)	C2—C1—H11	111.6
C11—C6—C7	119.04 (14)	N1—C1—H12	110.4
C5—C6—C7	119.46 (14)	C2—C1—H12	113.0
C5—N1—C1	120.85 (14)	H11—C1—H12	108.5
C5—N1—C4	127.13 (13)	N1—C4—C3	103.67 (14)
C1—N1—C4	110.08 (13)	N1—C4—H41	110.4
C6—C11—C12	129.25 (16)	C3—C4—H41	111.7
C6—C11—H111	115.4	N1—C4—H42	110.9
C12—C11—H111	115.3	C3—C4—H42	110.4
C5—C10—C9	122.08 (16)	H41—C4—H42	109.6
C5—C10—H101	118.2	C15—C16—H161	109.4
C9—C10—H101	119.7	C15—C16—H162	110.6
O4—C14—O3	124.77 (16)	H161—C16—H162	109.5
O4—C14—C12	112.45 (14)	C15—C16—H163	108.3
O3—C14—C12	122.77 (16)	H161—C16—H163	108.3
C6—C5—C10	116.84 (15)	H162—C16—H163	110.7
C6—C5—N1	124.27 (15)	C16—C15—O4	107.71 (16)
C10—C5—N1	118.89 (15)	C16—C15—H151	111.3
C6—C7—C8	120.77 (15)	O4—C15—H151	107.9
C6—C7—H71	119.6	C16—C15—H152	111.9
C8—C7—H71	119.6	O4—C15—H152	107.8
C7—C8—N2	119.31 (15)	H151—C15—H152	110.0
C7—C8—C9	120.95 (16)	C1—C2—C3	103.20 (15)
N2—C8—C9	119.74 (15)	C1—C2—H21	111.2
C8—N2—O2	119.06 (15)	C3—C2—H21	111.1
C8—N2—O1	118.37 (16)	C1—C2—H22	112.1
O2—N2—O1	122.55 (16)	C3—C2—H22	109.2
C14—C12—C11	122.22 (15)	H21—C2—H22	109.9
C14—C12—C13	113.25 (14)	C2—C3—C4	102.37 (16)
C11—C12—C13	124.43 (16)	C2—C3—H31	110.2
C12—C13—N3	178.06 (19)	C4—C3—H31	111.5
C8—C9—C10	119.72 (16)	C2—C3—H32	110.2
C8—C9—H91	119.1	C4—C3—H32	111.6
C10—C9—H91	121.2	H31—C3—H32	110.7
N1—C1—C2	103.93 (15)		

### Hydrogen-bond geometry (Å, °)

**Table:**

*D*—H···*A*	*D*—H	H···*A*	*D*···*A*	*D*—H···*A*
C15—H152···O2^i^	0.98	2.50	3.356 (2)	145
C16—H163···N3^ii^	0.98	2.60	3.574 (3)	172

Symmetry codes: (i) −*x*+1, −*y*+1, −*z*+1; (ii) *x*−1, *y*, *z*.
